# Triple Threat: Three Primary Malignancies Simultaneously Involving Three Genitourinary Organs

**DOI:** 10.1155/2023/3242986

**Published:** 2023-04-17

**Authors:** Katharina Mitchell, Reima El Naili, Lakshmikumar Pillai, Eric Mark Lopez, John Riordan, Wallis Marsh, Adam Luchey, Ali Hajiran

**Affiliations:** ^1^West Virginia University Department of Urology, Morgantown, WV, USA; ^2^West Virginia University Department of Pathology, Morgantown, WV, USA; ^3^West Virginia University Department of Vascular Surgery, Morgantown, WV, USA; ^4^Berkeley Medical Center Nephrology, Martinsburg, WV, USA; ^5^West Virginia University Department of Surgical Oncology, Morgantown, WV, USA

## Abstract

Statistically, the chance of having concurrent renal cell carcinoma (RCC), urothelial carcinoma of the bladder (UC), and a neuroendocrine tumor (NET) of the renal parenchyma is less than one in a trillion. Herein, we describe an unusual case of a 67-year-old female who presented with bilateral flank pain and severe gross hematuria. Cross-sectional imaging revealed two large heterogeneous, endophytic renal masses with a single enlarged paracaval lymph node. Diagnostic cystoscopy was performed for completion of gross hematuria evaluation and revealed a concurrent papillary bladder tumor. Percutaneous biopsies of bilateral renal masses revealed clear cell RCC involving the left kidney and well-differentiated NET involving the right kidney, and transurethral resection of the bladder tumor revealed high-grade nonmuscle invasive urothelial carcinoma. The patient elected to undergo bilateral nephroureterectomy, radical cystectomy, and retroperitoneal and pelvic lymphadenectomy. Final pathology confirmed the presence of three different malignancies: noninvasive high-grade papillary UC of the bladder (pTaN0), left renal clear cell RCC (pT2bN0), right renal well-differentiated NET, and a single paracaval lymph nodes positive for metastatic NET (pT2aN1).

## 1. Introduction

Bladder cancer is the fourth most common cancer in men and the fifth most common malignancy overall [1]. About 81,180 new cases of bladder cancer will be diagnosed in 2022 [1]. While less common than bladder cancer, clear cell renal cell carcinoma (RCC) is still relatively common with an overall incidence rate of 3.59 cases per 100,000 population [2]. However, in the literature, the occurrence of RCC with simultaneous urothelial carcinoma is rare, with around 50 cases reported total [3]. Even more seldom seen than either of the aforementioned malignancies in isolation are renal neuroendocrine tumors (NETs) which are exceedingly rare with only 100 described in the literature and an overall incidence of primary renal NETs being 0.13 per one million people [4, 5, 6]. Furthermore, primary neuroendocrine tumors of the kidney compile less than 0.4% of all NETs [6]. Here, we present the case of a patient found to have renal neuroendocrine cancer, clear cell RCC, and high-grade urothelial cancer concurrently. To the best of our knowledge, no such cases have been previously reported.

## 2. Case Presentation

**Figure 1:**
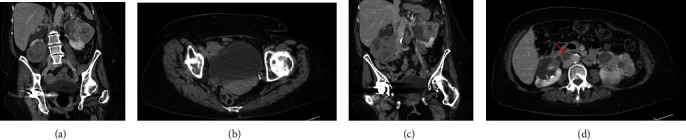
(a) Coronal imaging of large multilobular solid and cystic mass involving most of the left mid to upper pole with a portion of the mass wall not well delineated possibly involving the renal pelvis measuring approximately 8.5 × 9.2 × 9.2 cm. (b) Axial view of high-density material within the posterior aspect of the urinary bladder. (c) Coronal imaging of right complex solid and cystic mass in the upper pole measuring 2.4 × 1.7 cm, large solid cystic mass at the lower pole measuring 8.6 × 9.1 × 7.8 cm, and an ill-defined cortical based mass like area in the lower pole measuring approximately 17 × 9 mm. (d) Axial view of aorto-caval adenopathy with arrow pointing to periaortic adenopathy.

**Figure 2:**
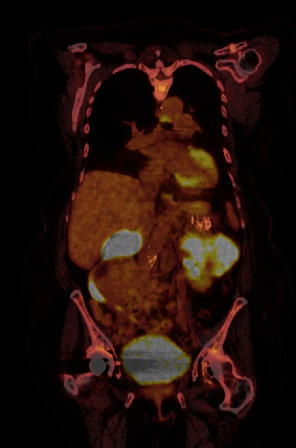
PET scan with right mass demonstrating moderate metabolic activity and left renal mass irregular with hypermetabolic activity.

**Figure 3:**
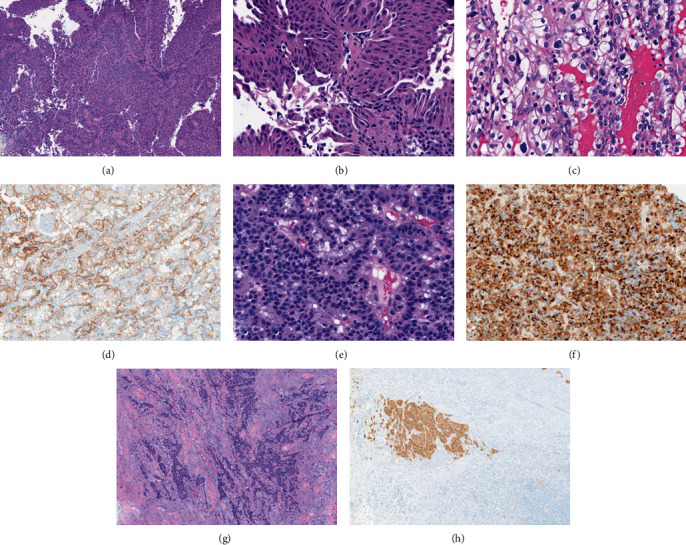
The histopathological findings of the radical cystectomy specimen showed a small focus of noninvasive high-grade urothelial carcinoma, composed of tumor cells with nuclear atypia, pleomorphism, and hyperchromasia ((a, b) hematoxylin and eosin, ×100 and ×400 magnification). The left radical nephrectomy specimen showed a clear cell renal cell carcinoma with nests of clear cells surrounded by intricately branching vascular septa and hyperchromatic nuclei ((c) hematoxylin and eosin, ×400 magnification). These tumor cells demonstrated membranous staining pattern with carbonic anhydrase IX immunohistochemical stain ((d) ×200 magnification). Histopathologic evaluation of the right nephrectomy specimen showed the typical nested pattern of a well-differentiated neuroendocrine tumor surrounded by fibrous stroma ((e) hematoxylin and eosin, ×400 magnification). Chromogranin A immunohistochemical stain showed diffuse granular staining in the tumor cells ((f) ×200 magnification). One out of three inter-aorto-caval lymph nodes showed metastatic neuroendocrine carcinoma ((g) hematoxylin and eosin, ×100 magnification), confirmed by synaptophysin immunohistochemical stain ((h) ×100 magnification).

A 67-year-old female with a past medical history of nicotine dependence, hypertension, chronic obstructive pulmonary disease, cardiomyopathy, and recurrent pyelonephritis as a child presented to the emergency department with severe left-sided back pain. She endorsed having heavy gross hematuria for a few weeks with no prior episodes. Family medical history included her mother's history of breast cancer and a history of kidney and lung cancer in her father. Workup included a computerized tomography angiography (CTA) with delayed phase imaging which shows a right complex solid and cystic mass in the upper pole measuring 2.4 × 1.7 cm, large solid cystic mass at the lower pole measuring 8.6 × 9.1 × 7.8 cm, and an ill-defined cortical based mass like area in the lower pole measuring approximately 17 × 9 mm. In the left kidney, she was seen to have a large multilobular solid and cystic mass involving most of the left mid to upper pole with a portion of the mass wall not well delineated possibly involving the renal pelvis measuring approximately 8.5 × 9.2 × 9.2 cm. Additionally, paracaval adenopathy was appreciated (Figure 1). Patient was then evaluated by a medical oncologist who ordered a positron emission tomography (PET) CT which showed the right mass to demonstrate moderate metabolic activity and the left renal mass to be irregular with hypermetabolic activity. There was no evidence of any additional disease outside of the patient's urinary tract, and the previously observed adenopathy did not enhance (Figure 2). In light of these findings, when the patient established care with our group, a discussion was regarding the high likelihood of need for bilateral nephrectomy due to size and location of bilateral renal masses. Given concern for the presence of upper tract urothelial carcinoma, we proceeded with percutaneous biopsy of bilateral renal masses to clarify histology. Additionally, due to history of gross hematuria, a diagnostic cystoscopy was also performed revealing a 3 cm papillary lesion on the right lateral bladder wall, just lateral to the right ureteral orifice with final pathology consistent with high-grade nonmuscle invasive urothelial carcinoma (cTa). An interventional radiologist was able to biopsy both renal masses percutaneously, and the results showed renal clear RCC from the left renal mass biopsy and well-differentiated NET from the right renal biopsy. The patient's case was reviewed at a multidisciplinary genitourinary tumor board, and the consensus was to offer bilateral nephroureterectomy with radical cystectomy and retroperitoneal and pelvic lymphadenectomy with curative intent. The patient, who was experiencing debilitating bilateral flank pain requiring narcotics and severe gross hematuria, elected to proceed with radical extirpative surgery with the understanding she would be dialysis dependent. A tunneled-cuff dialysis catheter was placed preoperatively. The patient was then taken to the operating room where she underwent open laparotomy via a midline incision, bilateral radical nephroureterectomy, retroperitoneal lymphadenectomy, pelvic lymphadenectomy, and anterior pelvic exenteration. Intraoperatively, the solitary enlarged paracaval lymph node was found to be densely adherent to the anterior wall of the inferior vena cava. Therefore, proximal and distal control of the inferior vena cava was established, and the lymph node was removed en bloc with a segment of the wall of the vena cava. The inferior vena cava was primarily repaired using running nonabsorbable monofilament suture. The patient tolerated the procedure well without any perioperative complications. Dialysis was initiated during her hospital admission, and she was discharged home on postoperative day five. Final pathology was consistent with noninvasive high-grade papillary UC of the bladder (pTaN0), left renal clear cell RCC (pT2bN0), right renal well-differentiated NET, and a single paracaval lymph nodes positive for metastatic NET (pT2aN1) (Figure 3). All surgical margins were negative. Patient is to begin treatment with somatostatin.

## 3. Discussion

Appropriate workup is of utmost importance for surgical planning in all scenarios but especially when presented with a patient with concern for multiple malignancies. Patients with gross hematuria warrant multiphasic cross-sectional imaging to evaluate both the renal parenchyma and the urothelium, using CT or magnetic resonance imaging (MRI) urography in addition to white light diagnostic cystoscopy. This case highlighted the importance of completing the full diagnostic workup with lower urinary tract endoscopic evaluation as concurrent bladder cancer could have easily been missed if the patient's hematuria had been attributed to having bilateral large infiltrative renal masses. Preoperative renal mass biopsy was also helpful in this case to help establish histologic diagnosis and rule out upper tract urothelial carcinoma, which may have altered the patient's treatment course to receiving neoadjuvant cisplatin-based chemotherapy prior to bilateral nephroureterectomy. Via this diagnostic approach, our patient was found to have RCC of the left kidney, well-differentiated NET of the right kidney, and high-grade nonmuscle invasive urothelial carcinoma (cTa) prior to radical surgical extirpation.

Certain types of renal tumors, such as oncocytoma, may be very challenging to differentiate from malignant renal masses [7]. As any surgical intervention to remove a renal mass appropriately will worsen renal function to some degree, understanding if bilateral renal surgical manipulation is truly warranted prior to time of surgery is a priority. Renal mass biopsy (RMB), per the American Urologic Association (AUA) guidelines, should be “obtained on a utility-based approach whenever it may influence management” [8]. Additionally one “should consider RMB when a mass is suspected to be hematologic, metastatic, inflammatory, or infectious” [8]. When obtaining a RMB on a solid renal mass, core biopsies are preferred over FNA. Studies have shown that core biopsy sensitivity and specificity are 99.1 and 99.7%, respectively, with complication rates between 8.5 and 10.4%, most of which were minor and self-limited [8]. Additionally, Yang et al. compared core biopsies directly to FNA showing the diagnostic rate, sensitivity, and diagnostic accuracy of 72%, 78%, and 96%, respectively, for FNA and 87%, 92%, and 94% for core biopsy [9].

RCC can be managed via various approaches depending on size, location, patient comorbidities, and patient preferences. When comparing partial to radical nephrectomy, oncologic success is similar for clinical T1a and T1b renal masses and select T2 renal tumors. When considering surgical approaches for RCC the RENAL nephrometry score is a highly useful particularly for patient counseling. The RENAL nephrometry score assigns numerical values to renal masses and attributes complexity grades and likelihood percentages of major complications based on the tumor radius, percentage endophytic vs. exophytic, nearness to the collecting system or sinus, anterior vs. posterior location, location relative to the polar lines, and whether the tumor has a hilar location touching the vein or artery [10]. With our patient's left renal RCC having a score of 11 h with 21.9% of major complications, a shared decision was made that radical nephrectomy would be most appropriate.

Neuroendocrine tumors are very rare, comprising around 2% of all malignancies [11]. These malignancies are more commonly seen in females 2.5 : 1 [11]. NETs can arise from any tissue or organ, even organs that do not normally have neuroendocrine cells [4]. Primary neuroendocrine tumors of the kidney compile less than 0.4% of all NETs [6]. Based on the review of renal NETs by Cleveland Clinic, horseshoe kidney (17.8%) and teratomas (14.3%) can be commonly found in patients with renal NETs. Many theories regarding the development of renal NETs exist including the following: NETs arising from primitive totipotential stem cells that differentiate in a neuroendocrine direction, metastasis from an undiagnosed primary tumor site to the kidney, activation of aberrant gene sequences in a totipotential stem cell line, and simultaneous renal congenital abnormalities [4]. NETs are divided into either well-differentiated (low grade to intermediate grade) neuroendocrine tumors or poorly differentiated (high grade) neuroendocrine carcinoma (NEC) based on their clinical behavior, histology, and proliferation rate [11]. While it has been typically seen that those with well-differentiated NETs fare better than those with poorly differentiated NETs, the low-grade/high-grade dichotomization for prediction of outcome is not a hard rule [11]. Some rapidly dividing cells of high-grade aggressive carcinomas can be potentially eradicated with multiagent chemotherapy, while the slow mitotic rate associated with low-grade NETs can make them more resistant to treatment [11]. The mainstay of treatment for low-grade tumors is surgical resection, with unresectable and symptomatic disease treated with somatostatin analogs and/or interferon-*α* [11]. For high-grade NETs/metastatic disease, etoposide/platinum-based chemotherapy is preferred [11]. When it comes to renal NETs for localized disease, nephrectomy and lymph node dissection is standard. However if for metastatic renal NENs, because there a no clinical trials to define optimal treatment for renal NENs at any stage, treatment is reflective of NET in other locations [12]. In a paper by Nguyen et al., 166 cases of primary neuroendocrine renal cell tumors were identified (grade 1 NET, grade 2 NET, large cell neuroendocrine carcinoma, and small cell neuroendocrine carcinoma), and a Kaplan-Meier survivor was performed which reported a 5-year OS of 50% and a 5-year DSS of 52% [12]. Additionally, they then performed a univariate analysis to determine how various factors contributed to overall and disease-specific survival which showed that large and small cell NECs were associated with the poorest OS and DSS [12]. As our patient was seen to have renal NEC, per previous papers, we performed nephrectomy and lymph node dissection. Based on the final pathology demonstrating well-differentiated renal NEC with 1/3 inter-aorto-caval lymph nodes positive for metastatic neuroendocrine carcinoma, because there are no clinical trials to define optimal treatment, the patient subsequently is being treated as standard for low-grade NEC and low-grade tumors with unresectable disease and therefore will begin somatostatin indefinitely. PROMID was a double-blind, randomized, controlled trial which compared octreotide with placebo and showed significantly improved time to progression in the octreotide group (14.3 vs. 6 months in the placebo group) in both functionally active and inactive NETs [13]. Patients in the PROMID trial were kept on octreotide throughout the trial from 2001 to 2008. There are no clinical trials to define optimal treatment for renal NENs at any stage [12], and trials are limited due to the rarity of this pathology; however, as the PROMID trial concluded, “individualized treatment recommendations taking into account risk factors for tumor progression, such as the proliferation rate and tumor load as well as the patient's wish, are warranted” [14].

High-grade cTa urothelial carcinoma of the bladder which is 3 cm is at the cutoff of the intermediate-risk/high-risk categories. These tumors typically would be managed with initial resection, second look resection 2-6 weeks later, with treatment with intravesical BCG. However, because in this case patient required bilateral nephrectomy, the decision was made to perform radical cystectomy in this patient.

This case report highlights the importance of appropriate workup for gross hematuria, which is culpable for the discovery of our patient's bilateral renal masses in addition to a bladder mass. Demonstrated nicely as well in this report is the key utilization of cystoscopy with biopsy of suspicious lesions/masses for the completion of a gross hematuria workup, as patient did indeed have pathologic evidence of lower urinary tract urothelial carcinoma. Finally, the utility of renal mass biopsy in surgical decision-making and patient counseling is vividly evident in this case as this very much informed out ability to better counsel the patient regarding options and prognosis.

NETs, urothelial cancer, and RCC all have been linked to various genetic mutations. When analyzing specific genes for mutation in NETs, there has been seen to be enrichment with mutations of TP53, CDKN1B, KRAS, MEN1, RB1, CREBBP, APC, DAXX, LPCAT2, and SETD2; deletions of TP53, CDKN2A, CDKN2B, CDKN1B, PTPRD, CBFA2T3, CAMTA1, ANKDR11, LINC00881, PRKN, and ZNF407; and amplifications of PCAT1/MYC and MDM2 [15]. Gene mutations are also commonly seen in bladder cancer and play an integral role in the transformation of urothelial cells into urothelial carcinoma, the most common genes seen to be involved including FGFR3, PIK3CA/AKT1, and TSC1/TSC2 [16]. Finally, to turn our attention RCC, while ccRCCs exhibit typically less than 20 DNA copy number alterations, proportionally, however, there are higher than anticipated copy number alterations involving whole chromosome arms [2]. The most common genetic alterations appreciated in ccRCC include inactivation of von Hippel-Lindau (VHL) gene (80%), Polybromo 1 (PBRM1) (45%), SET domain containing protein 2 (SETD2) (10-15%), and BRCA1-associated protein-1 (BAP1) (10-15%) [17].There may be a genetic link in the causation of these pathologies simultaneously, but our patient deferred genetic testing. As far as we are aware, this is the only case report in the literature which describes a patient afflicted with RCC, renal NET, and urothelial carcinoma concurrently.

## Data Availability

The data that support the findings of this case report are available from the corresponding author, Katharina Mitchell, upon reasonable request.
